# Supplementary material for: Magnetite-Supported Gold Nanostars for the Uptake and SERS Detection of Tetracycline
Source: Nanomaterials (Basel). 2018 Dec 27;9(1):31. doi: 10.3390/nano9010031 (PMC6359395; doi:10.3390/nano9010031)
Supplement: Supplementary file 1 [file nanomaterials-09-00031-s001.pdf]

Supplementary Material

Magnetite-Supported Gold Nanostars for the Uptake and SERS Detection of Tetracycline

Paula C. Pinheiro, Sara Fateixa *, Helena I. S. Nogueira and Tito Trindade

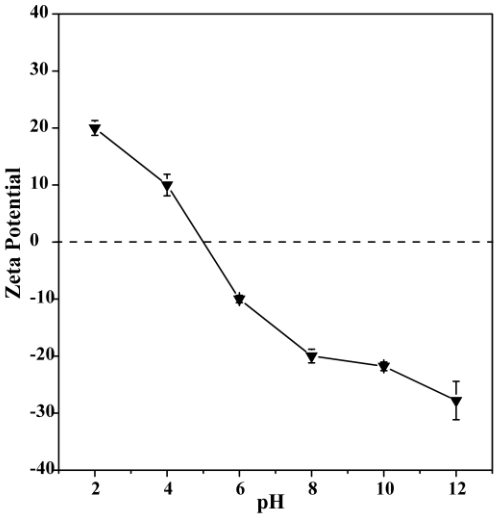

Figure S1. Zeta potential measurements of cubic shape magnetite particles in function of pH.

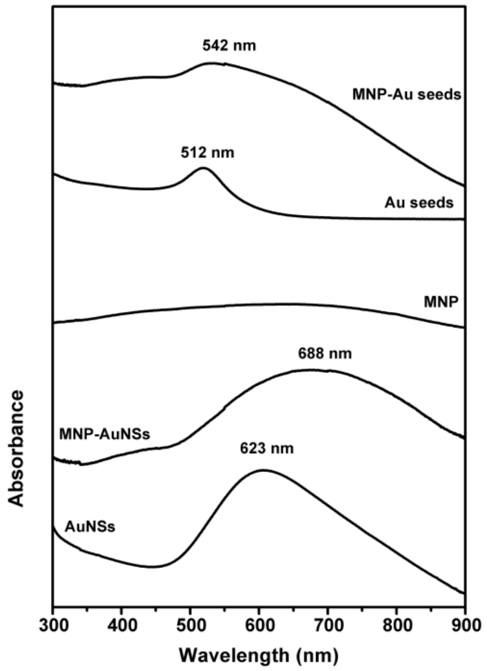

Figure S2. Optical spectra of MNP-Au seeds, Au seeds, MNP, MNP-Au NSs and Au NSs (supernatant).

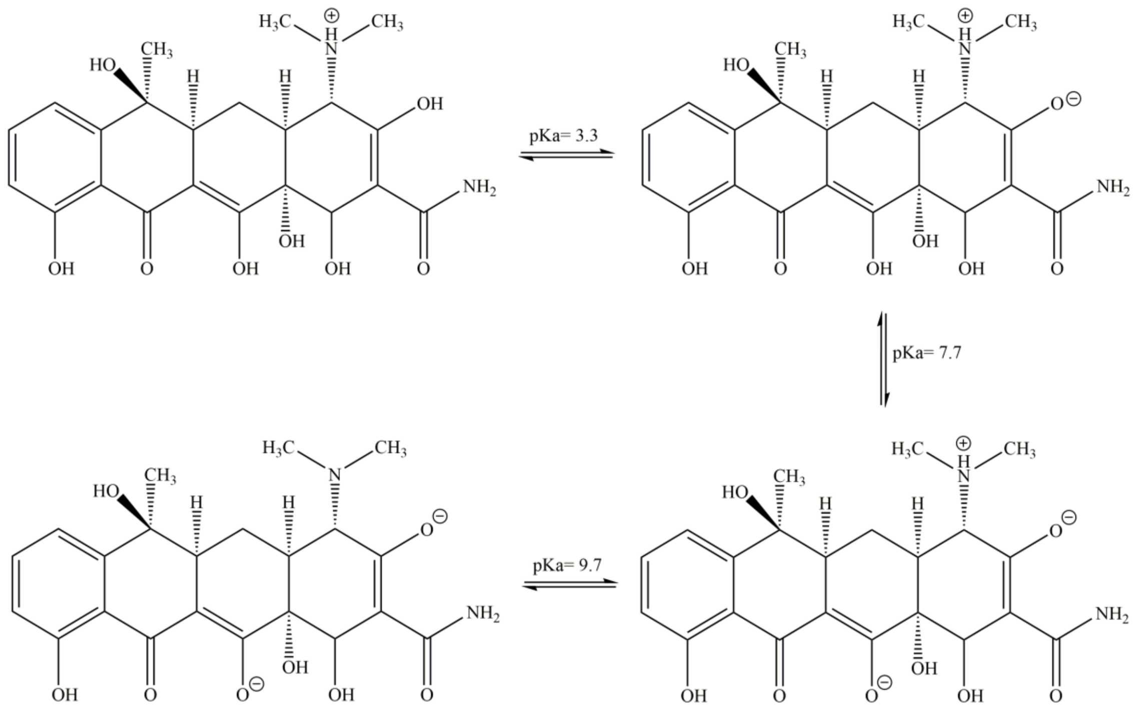

Figure S3. Structures and pKa values of tetracyclines.

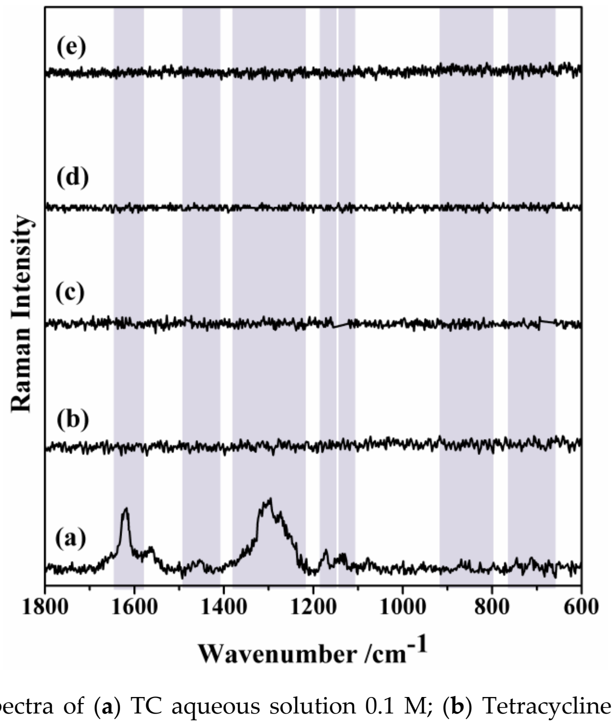

Figure S4. Raman spectra of (**a**) TC aqueous solution 0.1 M; (**b**) Tetracycline aqueous solution 10 μM; (**c**) Mag-AuNS solid substrate; (**d**) Fe_3O_4 nanoparticles after contact with TC for 20 min at initial concentration 10 μM; (**e**) Mag-AuSeeds nanoparticles after contact with TC for 20 min at initial concentration 10 μM.

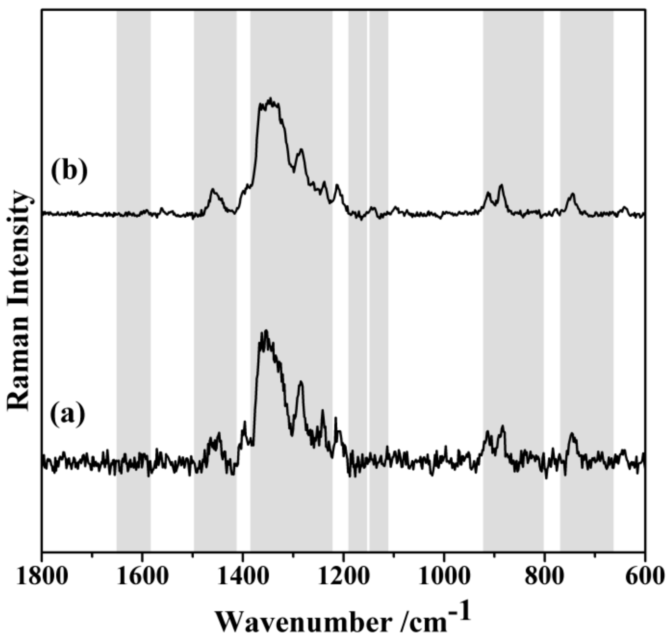

Figure S5. SERS spectrum for TC (10 μM) using Mag-AuNS (**a**) before and (**b**) after magnetic concentration (excitation at 633 nm, 0.2 mW laser power). Grey shadow: characteristic Raman bands for TC powder.

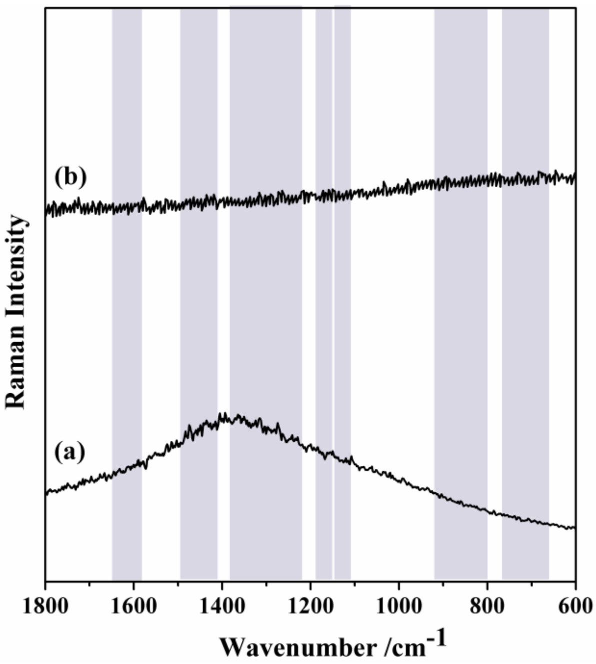

Figure S6. Average Raman spectra of Mag-AuNS substrate with (**a**) estuarine water from Aveiro lagoon (salty water) and (**b**) mineral water (excitation at 633 nm, 0.2 mW laser power, 22500 spectra, 0.1 s, 1 acquisition).

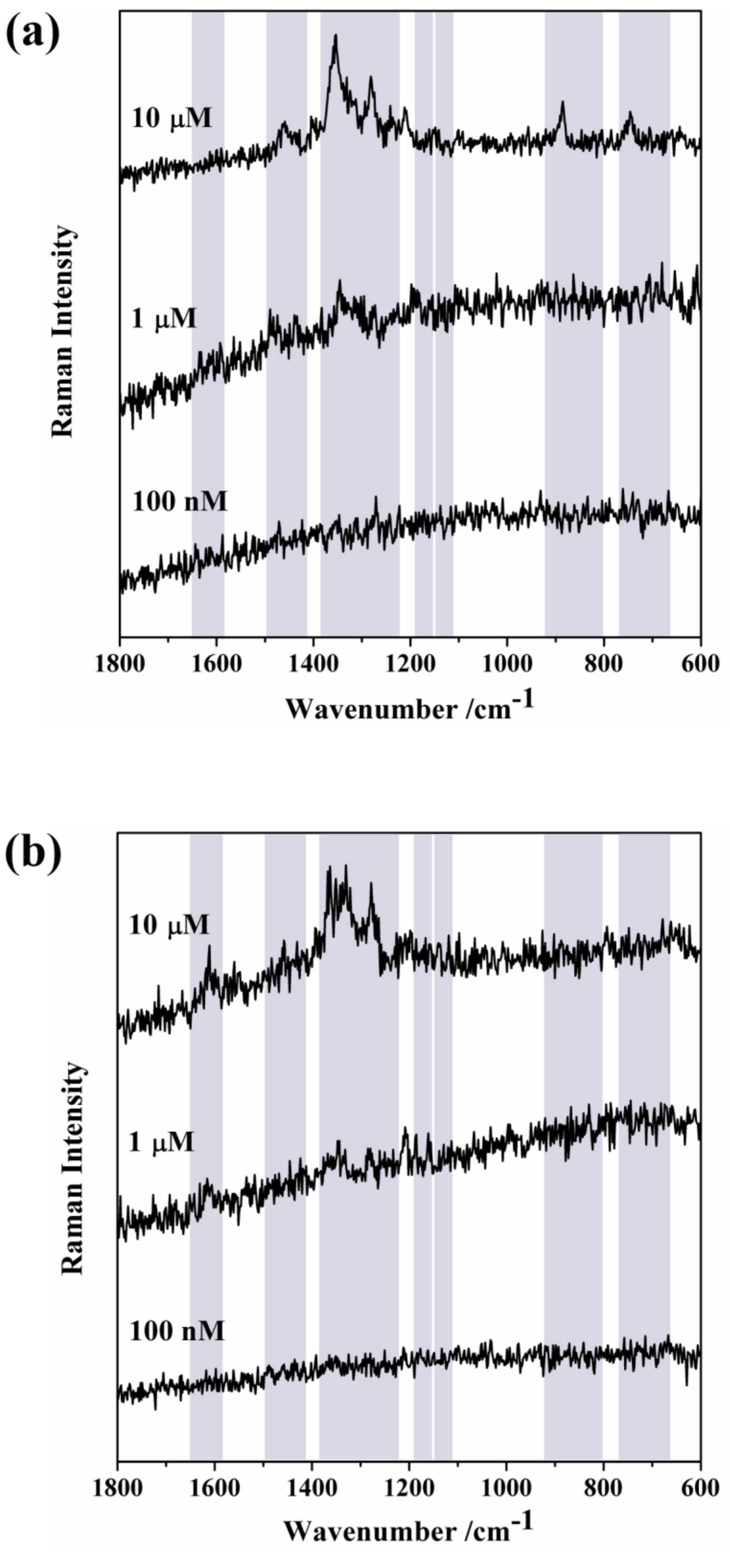

Figure S7. SERS spectra of TC at several concentrations using MNP-AuNS as the SERS substrate in (**a**) estuarine water from Aveiro lagoon and (**b**) mineral water.
